# Risk Factors in the Development of Very Late-Onset Schizophrenia-Like Psychosis: A Scoping Review

**DOI:** 10.1192/bjo.2024.145

**Published:** 2024-08-01

**Authors:** Isabelle Gallier-Birt, David White, Kaldora Ibekwe

**Affiliations:** 1 University of Warwick, Coventry, United Kingdom; 2 Coventry and Warwickshire Partnership Trust, Coventry, United Kingdom

## Abstract

**Aims:**

Very Late-Onset Schizophrenia-Like Psychosis (VLOSLP) is a condition resembling schizophrenia, which has a first onset in individuals at age 60 or later. Understanding the risk factors associated with the development of this condition is crucial, given the increasing ageing population and the elevated mortality rate in VLOSLP patients compared with the general population. This scoping review aims to explore and map the risk factors associated with VLOSLP development and begin to identify potential mechanisms linking these factors through comprehensive literature searching, screening and data extraction.

**Methods:**

Conducted as a scoping review; MEDLINE, Embase and APA PsycInfo were searched using the terms: “Very-Late Onset Schizophrenia-Like Psychosis”, “VLOSLP”, “Geriatric Psychosis” and “Geriatric Schizophrenia”. Inclusion criteria focused on psychosis with onset at 60 years or older and the identification of at least one potential risk factor. Studies were excluded which did not specifically refer to age of onset or concerned psychosis with an attributable organic cause. Thematic analysis was used to categorise risk factors into biological and psychosocial themes, followed by further organisation into specific subthemes.

**Results:**

Out of 326 initial results, 41 studies met inclusion criteria and underwent data analysis. Key risk factors included female gender, sensory impairment, social isolation, and migration, with potential interconnections identified between factors. Postulated mechanisms for the role of a risk factor in VLOSLP development recorded in the literature were included in the review. Mechanisms showed potential co-linkage between subthemes of risk factor. Migration status was also shown to impact gender as a risk factor, with male migrants experiencing higher rates of VLOSLP than their female counterparts. Thematic analysis highlighted how social isolation, a prominent risk factor, might be linked to, or reinforced by, sensory impairment, trauma, bereavement, and premorbid personality traits.

**Conclusion:**

The scoping review revealed that risk factors for VLOSLP span across biological, social, and psychological domains, with the findings contributing to the broader understanding of schizophrenia-like psychoses in the elderly population. Social isolation emerged as a widely-cited factor, reiterating the importance of managing risk factors for VLOSLP in vulnerable individuals via a holistic and multidisciplinary approach. Results bring attention to the bi-directional relationships between risk factors and psychotic illness, with perceived risk factors a potential consequence of the psychosis. In response to this, future work may involve large-cohort longitudinal studies to outline temporal relationships between risk factors and symptom development.

